# Systematic Analysis of microRNA Targeting Impacted by Small Insertions and Deletions in Human Genome

**DOI:** 10.1371/journal.pone.0046176

**Published:** 2012-09-25

**Authors:** Anindya Bhattacharya, Jesse D. Ziebarth, Yan Cui

**Affiliations:** 1 Department of Microbiology, Immunology and Biochemistry, University of Tennessee Health Science Center, Memphis, Tennessee, United States of America; 2 Center for Integrative and Translational Genomics, University of Tennessee Health Science Center, Memphis, Tennessee, United States of America; University of Navarra, Spain

## Abstract

MicroRNAs (miRNAs) are small noncoding RNA that play an important role in posttranscriptional regulation of mRNA. Genetic variations in miRNAs or their target sites have been shown to alter miRNA function and have been associated with risk for several diseases. Previous studies have focused on the most abundant type of genetic variations, single nucleotide polymorphisms (SNPs) that affect miRNA-mRNA interactions. Here, we systematically identified small insertions and deletions (indels) in miRNAs and their target sites, and investigated the effects of indels on miRNA targeting. We studied the distribution of indels in miRNAs and their target sites and found that indels in mature miRNAs, experimentally supported miRNA target sites and PAR-CLIP footprints have significantly lower density compared to flanking regions. We identified over 20 indels in the seed regions of miRNAs, which may disrupt the interactions between these miRNAs and their target genes. We also identified hundreds of indels that alter experimentally supported miRNA target sites. We mapped these genes to human disease pathways to identify indels that affect miRNA targeting in these pathways. We also used the results of genome-wide association studies (GWAS) to identify potential links between miRNA-related indels and diseases.

## Introduction

MicroRNAs (miRNAs) are short non-coding RNAs that function as post-transcriptional regulators of genes, repressing mRNA translation and causing mRNA decay [1]. Initial miRNA transcripts are processed in the nucleus to produce ∼100 nt long hairpin precursors, which are exported to the cytoplasm and processed into ∼22 nt long mature miRNA sequences that act on their mRNA targets within the RNA-induced silencing complex (RISC) [1]. MicroRNA target recognition is highly dependent on interactions between complementary sequences in miRNA seed regions and target sites in mRNAs [2], [3], [4], [5]. Therefore, miRNA targeting and function can be affected by sequence polymorphisms in miRNAs and their target sites. Over the last several years, several association studies have identified polymorphisms in miRNA genes and their target sites that are linked with risk for several diseases, including schizophrenia [6], nonsyndromic progressive hearing loss [7], cancer [8], [9], [10], [11], [12], [13], [14], [15], Parkinson’s disease [16], and stroke [17]. While experimental evidence providing a direct, functional role for these polymorphisms in disease development remains weak for most of these associations, some recent experiments have investigated how disease-associated polymorphisms impact the expression or function of miRNAs [6], [7], [8], [9], [10], [11], [12], [13], [14], [15]. For example, a single nucleotide polymorphism *rs2910164* in the pre-miRNA of miR-146a has been associated with increased risk for several types of cancer [8], [9], [10], [11], [12]. To supplement these association study results, further experimental investigation of miR-146a and *rs2910164* has shown that the polymorphism results in variation of expression of miR-146a [8], [9], [10], [11], [12] and its targets [18], including *BRCA1* [8], and that miR-146a promotes cell proliferation and colony formation in the NIH/3T3 cell line [10]. In another study, a “TTCA” deletion (*rs3783553*) in the 3′ UTR of *IL1A*, a gene that induces antitumor cell immunity, was shown to be associated with hepatocellular carcinoma risk, and subsequent experiments showed that the deletion enhances binding of miR-122 and miR-378 to *IL1A*, reducing its in vivo expression [15].

As links between miRNA-related polymorphisms and human diseases have been identified, there has been increasing interest in systematic evaluations of polymorphisms within miRNAs and their target sites. Saunders et al. performed one of the first analyses of polymorphisms in miRNAs and found that there was a relatively low level of variation within miRNAs compared to surrounding regions or miRNA target sites [19]. The low level of variation in miRNAs, particularly within mature miRNA sequences and miRNA seed regions, has been subsequently confirmed by further investigation of polymorphisms in humans [20], [21] and targeted sequencing of miRNAs in Arabidopsis [22]. Bao et al [23] developed a database, PolymiRTS, to systematically characterize SNPs in microRNA target sites and link them with complex traits. This database has been recently updated to integrate new data, including SNPs in miRNAs, experimentally supported miRNA target sites, and the results of genome-wide association studies (GWAS) of human diseases [24].

With the recent advances in sequencing technologies, there has been a rapid increase in the number of small indels identified in the human genome [25], [26], [27]. Genome wide analysis has shown that indels are the second most common type of genetic variants after SNPs and that they comprise approximately 18% of known variants [26], [28]. While previous analysis of polymorphisms has focused mainly on SNPs and a handful of indels within miRNAs and their target sites that have been identified [19], [22], a full investigation of how these newly identified indels may impact miRNA function has yet to be performed. There has also been a rapid growth in the number of experimentally supported miRNA target sites in humans [29], reducing the reliance on computational methods for miRNA target prediction. To a large extent, this growth is due to recent experiments, designated HITS-CLIP [30] and PAR-CLIP [31], that identified the specific mRNA sequences that interact with miRNAs in Ago protein-RNA complexes in the mouse brain and human embryonic kidney cells (HEK294), respectively. While large scale evaluation of miRNA targeting based on these experiments is still incomplete because they have only been performed for two specific cell and tissue types, the experiments have greatly expanded the number of experimentally determined miRNA target sites. Additionally, there have been a significant number of miRNA targets identified by low-throughput experiments utilizing, for example, luciferase reporter assays [32].

Here, we created a comprehensive collection of indels in miRNAs and their target sites in the human genome and analyzed the potential functional impact of these indels. We determined polymorphic miRNAs that have been previously associated with the risk of diseases and investigated how indels in miRNA target sites may alter miRNA regulation in human disease pathways. We also identified potential links between the indels altering miRNA targeting and human diseases using the results of association studies.

## Results

### Distribution of INDELs in 3′ UTRs and miRNAs

We collected all indel variations in dbSNP (build 135) that were located within either miRNAs or the 3′ UTRs of genes, the genetic regions that are believed to harbor the majority of functional miRNA target sites. Among the ∼6 million indels in dbSNP (build 135), 181 indels were located within 124 pre-miRNAs, including 51 in the mature sequence of 43 miRNAs and 26 in the seed region of 22 miRNAs. Additionally, 56,724 indels were located within 3′ UTRs of 9,420 genes, potentially affecting miRNA binding to these genes. Following the classification system provided by Mills et al [25], indels were categorized into three classes, namely (i) single-base-pair indels, (ii) repeat expansions consisting of repeated sequences of one or more nucleotides, and (iii) an “other” class of multiple-base-pair indels other than repeat expansions. The majority of indels within both miRNAs (55%) and 3′ UTRs (57%) were single-base-pairs, while there were fewer repeat expansions (10% of indels within miRNAs, 14% of indels in 3′ UTRs) and indels in the “other” class (35% of indels within miRNAs, 28% in 3′ UTRs). The distribution of indels in 3′UTRs and miRNAs was similar to the distribution across the entire genome (Figure S1).

**Figure 1:**
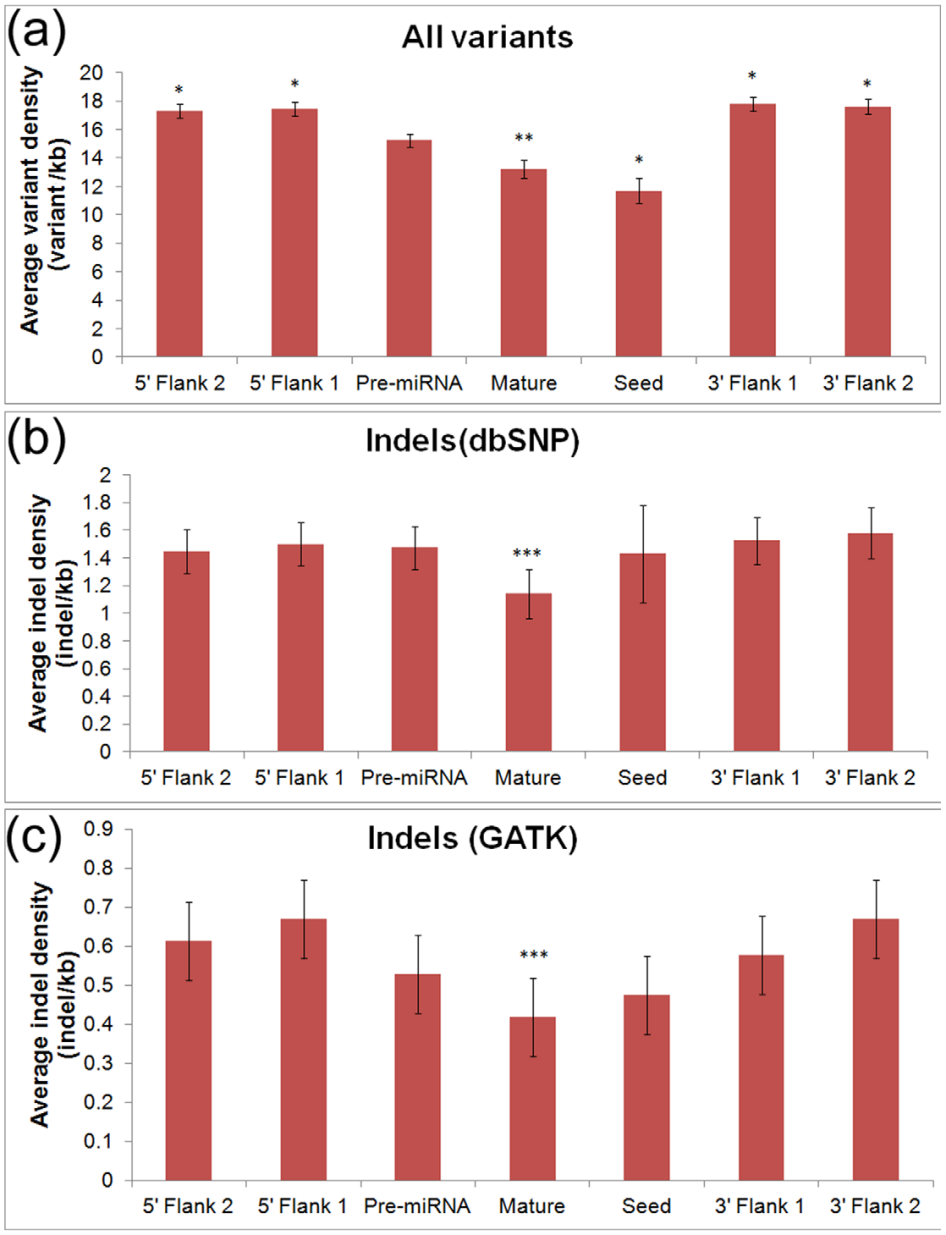
Density of all genetic variants (a) and indels (b) in dbSNP 135 as well as indels (c) from the GATK resource bundle in pre-miRNAs, mature miRNAs, miRNA seed regions, and flanking regions. Flanking regions 1 and 2 represent successive sequences adjacent to pre-miRNAs that were equal to the length of the pre-miRNA (∼100 bp). Error bars indicate the standard error. The density of all genetic variants (a) in pre-miRNAs was significantly different from the density in flanking regions, mature miRNAs, and seed regions (*p<0.01, **0.01<p<0.05). The density of indels in mature sequences (b) and (c) was significantly different than the density calculated by averaging across all four flanking regions (***0.01<p<0.05).

**Figure 2:**
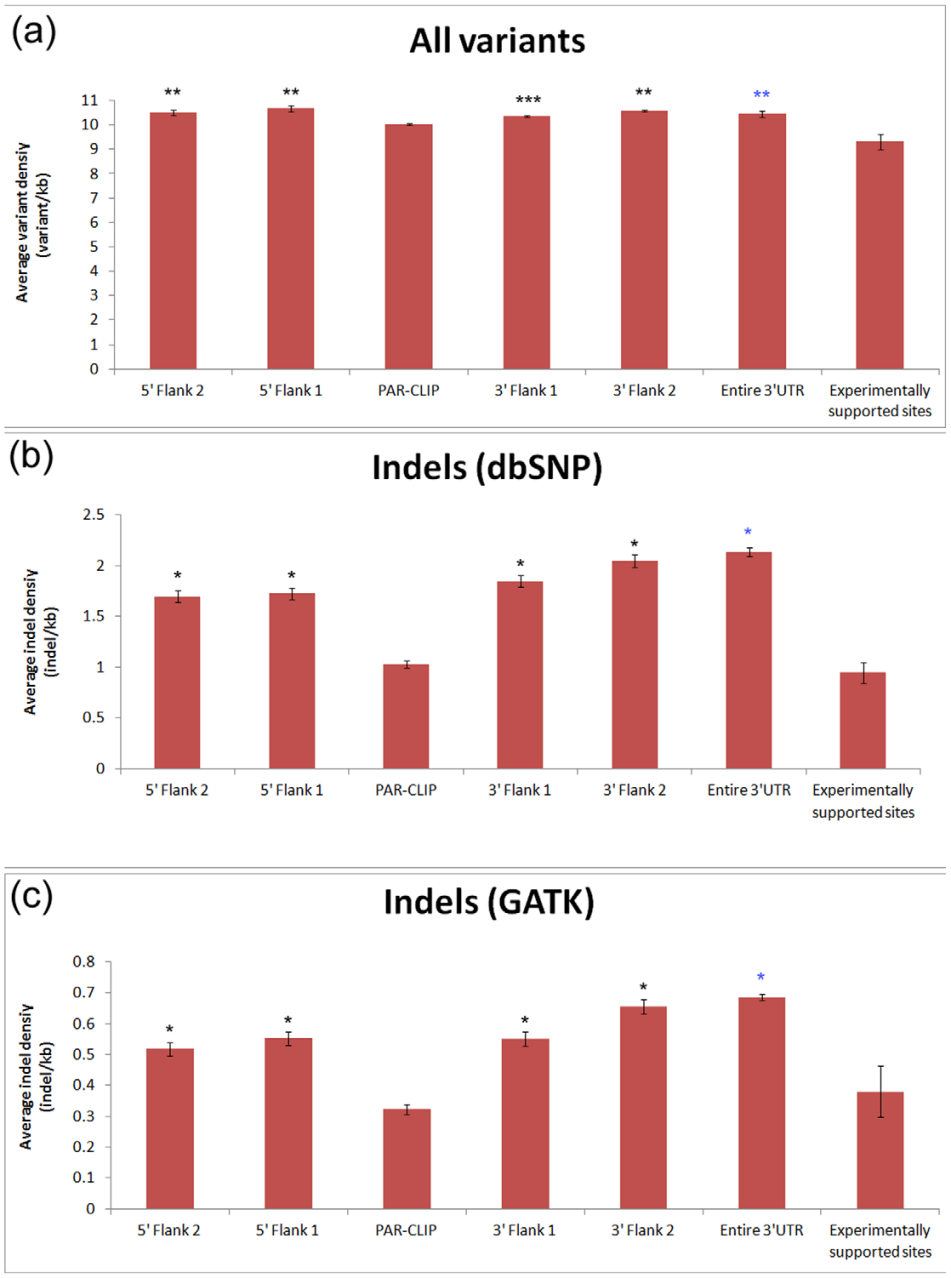
Density of all genetic variants (a) and indels (b) in dbSNP 135 as well as indels (c) from the GATK resource bundle in PAR-CLIP footprints and flanking regions, entire 3′ UTR and experimentally validated target sites. Flanking regions 1 and 2 represent successive sequences adjacent to PAR-CLIP footprints that were equal to the length of the footprints (∼41 bp). Error bars indicate the standard error. The density of all genetic variants (a) in PAR-CLIP footprints was significantly different from the density in flanking regions (**10^−5^<p<10^−3^, ***p = 0.04). The density of indels in PAR-CLIP footprints (b) and (c) was significantly different from the density in flanking regions (*p<10^−12^). The density of all genetic variants (a) in experimentally validated targets was significantly different from the density in entire 3′ UTR regions (**p = 5.7×10^−7^). The density of indels in experimentally validated targets (b) and (c) was significantly different from the density in 3′ UTR regions (*p<10^−12^).

MicroRNAs and their target sites have been shown to have lower polymorphism density than their surrounding regions [19], [21]. Furthermore, the level of variation within mature miRNA sequences and their seed regions has been found to be lower than that in complete precursor miRNA sequences. To determine whether indels have a similar distribution pattern in these regions, we calculated the average density for indels and all types of polymorphisms both within miRNAs and in the genomic regions flanking miRNAs (Figure 1). The average density of all polymorphisms matched the distribution patterns that have been found previously [19], [21] (Figure 1a); the density of all polymorphisms in mature and seed sequences is significantly lower than the polymorphism density within pre-miRNAs, while the density of flanking regions was significantly higher than that of the pre-miRNAs. In contrast, the density of indels in miRNAs compared with flanking regions does not show the same pattern (Figure 1b), as the indel density in pre-miRNAs is similar to the indel density in miRNA seed regions and flanking regions. For further investigation of this finding, we collected a set of high-confidence indels data from the GATK resource bundle (described in the Materials and Methods section) and identified their density in miRNAs (Figure 1c). Several features observed for the indel density within miRNAs and their flanking regions for the GATK indels (Figure 1c) were similar to those found using all indels in dbSNP (Figure 1b). However, we did find that the density of indels in the miRNA seed regions identified from the GATK data, while not as low as the density of indels in the entire mature sequences, was slightly lower than the indel density of flanking regions. One significant feature was maintained for the density of indels from dbSNP and GATK as well as the density of all polymorphisms, as the polymorphism density was significantly lower in mature miRNAs than in the flanking regions. We also investigated the density of indels in the miRNA target sites. We calculated the average density for indels and all types of variants in experimentally supported miRNA target sites, the entire 3′UTRs, PAR-CLIP footprints and their flanking regions (Figure 2). We found that the density of indels (both dbSNP and GATK indels) in PAR-CLIP footprints and experimentally supported miRNA target sites are significantly lower than those of flanking regions and entire 3′UTRs (p<10^−12^). The density distribution of all variants has a similar distribution pattern, but with less significant p-values (Figure 2a). These observations could suggest that there is selective pressure against genetic variants in miRNAs and their target sites and the selective pressure against indels in miRNA target sites is particularly strong (Figure 2b and Figure 2c).

### Indels in miRNAs

Polymorphisms within miRNAs have been shown to alter miRNA mediated gene regulation and contribute to disease pathogenesis [8], [9], [10], [11], [12], [18]. They may impact miRNA function through two main mechanisms, by either altering miRNA biogenesis and expression or by affecting the binding of the miRNA to its mRNA targets, potentially disrupting the targeting to its original targets and creating a new set of targets. While polymorphisms throughout the entire miRNA precursor sequence are likely to act through only the first mechanism, those within mature miRNA sequences, particularly within miRNA seed regions, can change miRNA function through both mechanisms. In total, we identified 144 miRNAs containing indels, including 25 miRNAs with indels in the seed region. Many of the miRNAs that contain indels in their precursor sequence have been previously linked with diseases. We identified indels in the pre-miRNA sequences of several miRNAs that have been linked to cancers. For example, the precursor of miR-558, which has been previously linked with aggressive neuroblastoma [33], contained 5 indels, and an indel (*rs34385807*) is located the pre-miRNA sequence of miR-141, which is involved in cancer proliferation [34], [35], [36], [37] and has been shown to target the tumor suppressor *PTEN* [38]. Additional precursor sequences of miRNAs containing indels include miR-520h [39], [40], [41], miR-486 [42], miR-489 [43], miR-223 [44], miR-373 [45], miR-630 [46] and miR-1233 [17], which have been shown to be involved in cancer development, and miR-631, which is associated with risk of coronary artery disease [47].

We also identified all experimentally supported targets of the miRNAs containing indels by searching PAR-CLIP results [31] as well as experimental targets contained in miRecords [48], TarBase 5.0 [49] and miTarBase [50] (Table S1). We are most interested in the potential functional impact of indels within mature miRNA sequences and seeds and will focus our discussion only on miRNAs with indels in their mature sequences. The mature sequence of miR-940, which has been shown to target the signaling gene *SEMA3F* [21], contains an indel (*rs3536504*) that may disrupt the binding of miR-940 to *SEMA3F* and other targets. Indels within miRNA seed regions may be particularly deleterious as complementarity between this region and the mRNA target is crucial for miRNA target recognition. One miRNA which has an indel in its seed region is miR-513a-1, a miRNA known to post-transcriptionally regulate *B7-H1* [51]. This indel, the single nucleotide insertion *rs35027589*, may disrupt the targeting of *B7-H1* by miR-513a and have downstream effects on the *B7-H1/PD-1* pathway, a critical pathway for modulating immune responses to cancer [52]. Similarly, miR-562 contains an indel, the 18 bp deletion *rs140596642*, removing a large portion of the miRNA including the seed region, which may play a critical role in the development of Wilms’ tumor by both causing increased expression of miR-562 and dysregulation of its targets including *EYA1* [53]. Another indel, the five nucleotide deletion *rs138461304* in the seed region of miR-559, may disrupt targeting of *ERBB2* by miR-559 [54], resulting in overexpression of *ERBB2*, an abnormality that has been associated with cancer [54], [55]. A four nucleotide long deletion indel included in the GATK resource bundle is located in the seed region of miR-302c, potentially disrupting regulation of its targets which include *ESR1* [56] and *CCND1* [57]. A previous study has associated targeting of ESR1 by miR-302c with a role in breast cancer [56]. Another investigation established that miR-302 simultaneously suppressed both the cyclin E-CDK2 and cyclin D-CDK4/6 pathways to inhibit human pluripotent stem cell tumorigenicity [57].

### Indels in miRNA Target Sites

To determine indels that may impact experimentally supported miRNA target sites, we analyzed data from two sources: mRNA sequences that have been shown to interact with miRNAs in PAR-CLIP experiments and miRNA:mRNA target pairs in miRecords [48], miTarBase [50], and TarBase 5.0 [49]. The PAR-CLIP experiments provide the specific mRNA sequences that are targeted by miRNAs and we, therefore, identified all indels that were located in PAR-CLIP footprint regions. Typically, polymorphisms that alter complementary between the mRNA target and the seed region of the miRNA are believed to be the most deleterious, and we therefore determined how indels within the PAR-CLIP footprints alter this complementarity. In total, 152 indels were located within the PAR-CLIP footprint regions, and each indel disrupted or created at least a 6mer match to a miRNA seed (Table S2). In contrast with PAR-CLIP, most low-throughput methods used to identify miRNA target sites only provide miRNA:mRNA target pairs that interact, not the specific target sequences. Therefore, to identify indels that alter this type of experimentally determined target, we collected 4,074 known mRNA:miRNA target pairs from the sources listed above. We then identified all indels in the 3′ UTR of each mRNA in these pairs and scanned the sequence surrounding these indels to determine if they disrupted or created a 6mer or longer sequence complementary to the seed region of the targeting miRNA. We found that 197 experimentally identified mRNA:miRNA pairs had a putative target site that was altered by an indel ().

### Integrated Analysis of SNPs and INDELs Altering miRNA Targeting in Disease Pathways

**Figure 3:**
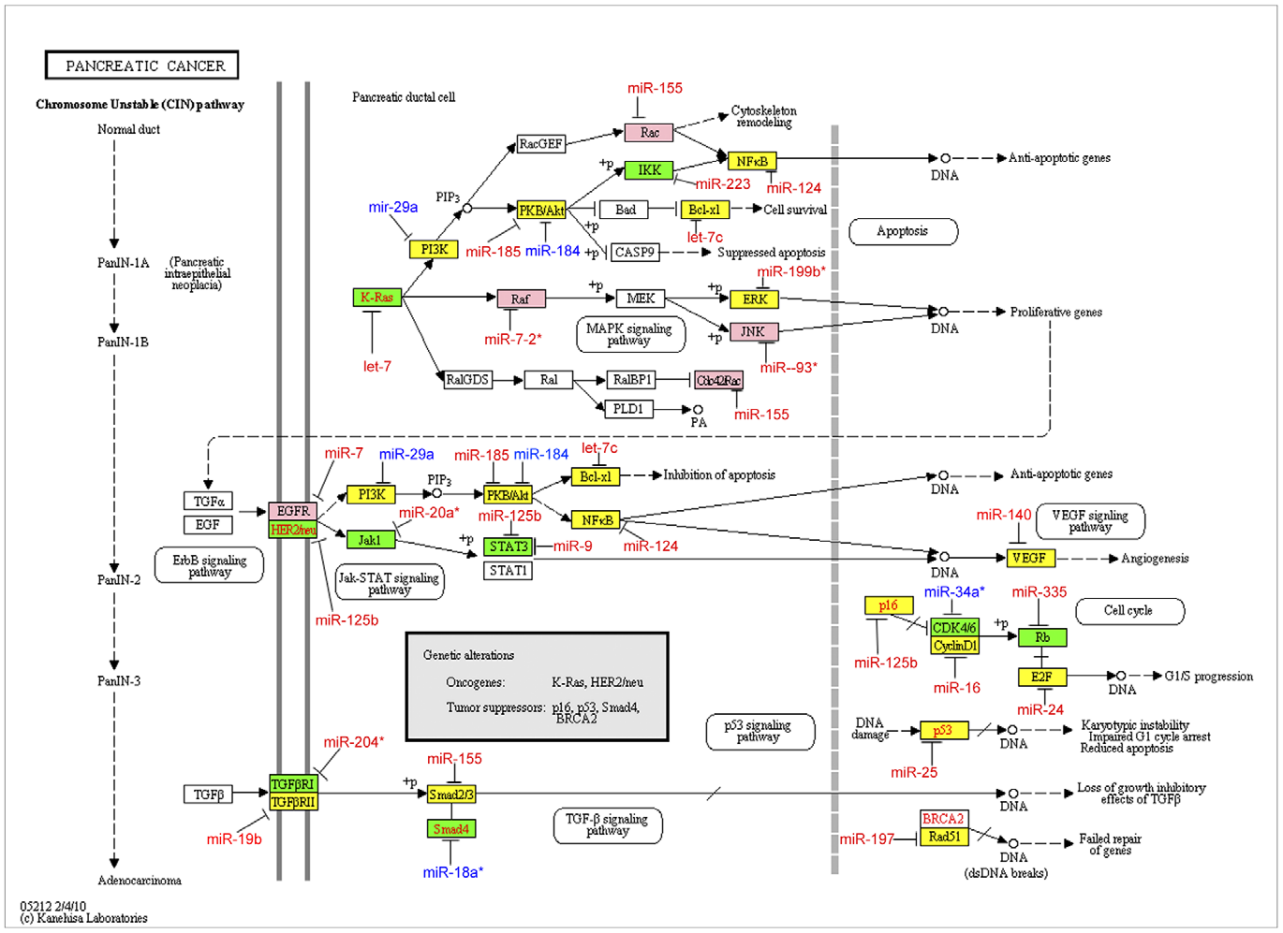
Genes in the pancreatic cancer pathway containing SNPs and indels that altered experimentally supported target sites. Genes containing only indels (pink), only SNPs (yellow), and both SNPs and indels (green) in target sites are within colored rectangles. The miRNAs that have been shown to target these genes are shown with red text for disrupted sites and blue text for created sites.

We found indels (Table S2 and S3) in experimentally supported miRNA target sites of 213 genes. To investigate the functional impact of these indels, these 213 genes were mapped to human disease pathways in the KEGG database [58]. Five pathways with ten or more genes with indels in miRNA target sites were selected for further analysis. (Table 1 and Table S4). We also identified SNPs in experimentally supported target sites of the genes in these pathways. Table 1 summarizes the number of genes with indels and SNPs in experimentally supported target sites in each pathway. Figure 3 shows genes containing indels, SNPs, or both in experimentally supported miRNA target sites, along with the miRNAs that target these genes, in the pancreatic cancer (hsa05212) pathway. For example, an indel (*rs78669011*) is located in the 3′ UTR of *EGFR* that disrupts a site complementary to the seed of miR-7. The targeting of *EGFR* by miR-7 has been experimentally supported by several experiments [43], [44] and both the gene and miRNA are known to play a role in cancer [59]. Similar figures for the pathways in cancer (hsa05200), prostate cancer (hsa05215), colorectal cancer (hsa05210) and the ErbB signaling (hsa04012) pathway are shown in Figures S2,S3,S4,S5.

**Table 1:** Selected human disease pathways containing genes with indels and SNPs in miRNA target sites.

KEGG Pathway	Number of geneswith indels	Percentage of geneswith indels	Number of geneswith SNPs	Percentage of geneswith SNPs
Pathways in cancer	25	7.62	77	23.48
Pancreatic cancer	13	18.57	26	37.14
Prostate cancer	12	13.48	28	31.46
Colorectal cancer	11	17.74	24	38.71
ErbB signaling pathway	11	12.64	15	17.24

### Identifying Potential Links between Indels that Alter miRNA Targeting with Human Diseases Using Results from Association Studies

Genome-wide association studies have identified a large number of genomic locations harboring genetic variants associated with various diseases. We attempted to integrate the results of these association studies with indels that impact miRNA targeting. We identified all indels in miRNA sequences or experimentally supported target sites that were located within linkage disequilibrium (LD) blocks of any high scoring markers associated with human diseases and traits from GWAS results collected in dbGaP and the NHGRI GWAS Catalog [60] (Table 2).

**Table 2:** Indels in miRNAs and miRNA target sites in linkage disequilibrium block for high-scoring markers from association studies.

Indel	Location	miRNA or Target Gene	GWAS Marker, p-value and Location	LD block boundaries: Left, Right	Disease/trait
rs34922018	Chr2:25166699	DNAJC27	rs713586, 6×10^−22^, 25158008	25150296, 25182193	Body Mass Index
rs35589685	Chr5:110464398	WDR36	rs2416257, 1×10^−6^, 110435490	110404185, 110467499	Plasma eosinophil count
rs34611972	Chr2:97498908	CNNM3	rs9948, 6×10^−6^, 97500800	97489870, 97525099	Erectile Dysfunction
rs34621455	Chr1:113213486	CAPZA1	rs17030613, 8×10^−6^, 113190807	113110548, 113234456	Blood Pressure
rs71737257	Chr3:12625276	RAF1	rs3729931, 7×10^−7^, 12626516	12624070, 12626516	Cardiomegaly
rs71717337	Chr3:12625275	RAF1	rs3729931, 7×10^−7^, 12626516	12624070, 12626516	Cardiomegaly
rs5745925	Chr15:75645967	miR-631	rs8028182, 3×10^−6^, 75718669	75632867, 75815758	Sudden cardiac arrest

**Figure 4:**
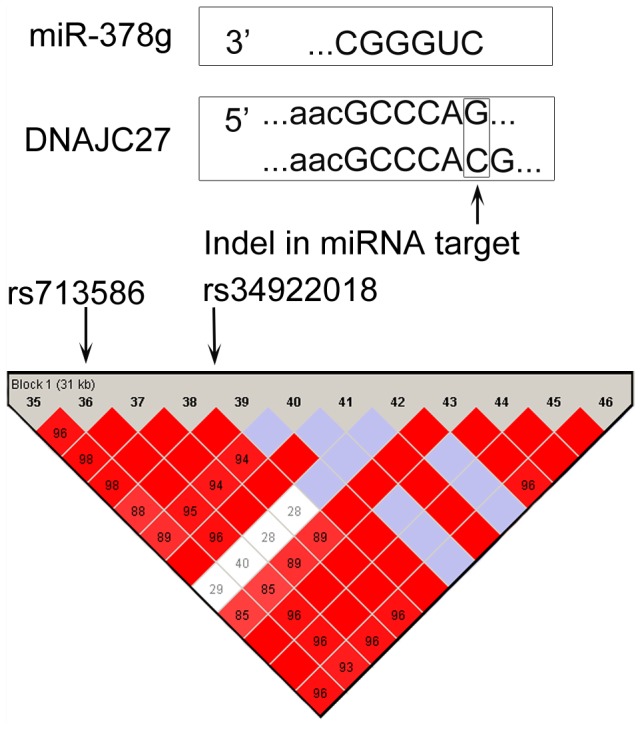
Disruption of a target site of miR-378g in the 3′ UTR of *DNAJC27* by an indel *rs34922018* that is in linkage disequilibrium with a high-scoring marker *rs713586* from a body mass index study.

Figure 4 shows an indel in a PAR-CLIP footprint region (*rs34922018*) that is within a LD block with the GWAS marker *rs713586*, which was found to have a significant association with “Body Mass Index” (*p* = 6×10^−22^) in a Genetic Investigation of Anthropometric Traits (GIANT) study of over 249,796 individuals of European ancestry [61]. This indel, within *DNAJC27*, may disrupt a binding site for miR-378g, resulting in dysregulation of the gene. Similarly, the indel *rs5745925* in miR-631 is within the same LD block as the association study marker SNP *rs8028182* in the CEU population. *rs5745925* is an insertion of CT that has been found to have a frequency of 0.93 in 90 individuals selected for individual screening in the NIH Polymorphism Discovery Resource (NIHPDR) [62]. The marker SNP *rs8028182* was found to be associated with sudden cardiac arrest in patients with coronary artery disease [63]; *SULT1A1*, an experimentally validated target of miR-631 [47], has been associated with the risk factor of coronary artery disease [64].

## Discussion

Recent advances in genome sequencing and association studies, as well as knowledge of disease pathways, have provided the resources to understand the impact of genetic variations that affect miRNA function. It has become possible to identify genetic polymorphisms altering miRNA targeting through large scale data integration. Although indels constitute the second most abundant type of genetic variants, there has not been a systematic analysis of indels in miRNAs and their target sites. In this work, we integrated multiple types of data to identify indels in miRNAs and in experimentally supported miRNA target sites and investigated the impacts of indels on miRNA targeting and function.

Previous investigations of genetic variations that impact miRNA targeting have mainly relied on computational algorithms for predicting miRNA target sites due to the lack of experimental data. These algorithms predict miRNA target sites based on complementarity between miRNA seeds and the target site, along with additional criteria including evolutionary conservation of binding sites [65], structural accessibility [66], or the nucleotide composition of the region containing the target [67]. However, these computational predictions are limited by high false positive rates and by the difficulty in assessing their false negative rates [68]. We, therefore, limited our analysis to those experimentally supported target sites, an approach that is becoming feasible mainly due to the recent advances in high throughput miRNA target identification such as PAR-CLIP experiments [31].

The integrated analysis of indels altering miRNA targeting and human disease pathways and GWAS results provided insights into the functional impacts of these genetic variants. We found that many genes in cancer pathways contain indels altering experimentally supported target sites (Figure 3, Figures S2,S3,S4,S5 and Table S4). For example, conserved helix-loop-helix ubiquitous kinase (*CHUK*), which is also known as *IKK1*, a protein kinase that plays an important role in pancreatic cancer and prostate cancer by regulating the *NF-kB* transcription factor, has two indels that may disrupt targeting by miR-223; *RB1* contains one indel in target site of miR-335; *STAT3* contains two indels in target sites of miR-9 and miR-125b; and *FOXO1* and *EFGR* have indels in target sites of miR-9 and miR-7 respectively. We further extended our analysis by integrating the indels altering miRNA targeting with the results of GWAS. Several miRNA-related indels were in linkage disequilibrium with high scoring markers of GWAS, including an indel *rs34922018* in linkage disequilibrium with high scoring marker *rs713586* from a body mass index study. The indel *rs34922018* found to disrupt a target site of miR-378g in the 3′ UTR of *DNAJC27* by an insertion of ‘*C*’ nucleotide (Figure 4).

## Materials and Methods

### INDELs in Pre-miRNA and Flanking Regions

The genomic locations of all miRNAs were obtained from miRBase release 18 [69]; the locations of pre-miRNAs were obtained from the genome coordinates file, while the locations of mature miRNAs were obtained from the ftp download section of miRBase. The start and end locations of 3′ and 5′ flanking regions around the pre-miRNA were determined by, depending on its transcriptional orientation, either adding or subtracting the length of each pre-miRNA from its location. All variants and only indels within miRNAs and their flanking regions were collected from dbSNP build 135 by setting the appropriated filter functions in the UCSC table browser [70], [71]. We also collected indels from the GATK resource bundle [72], a collection of standard files for working with human resequencing data, which includes a set of high-confidence indels for use with local realignment. Variants in miRNAs and flanking regions were then identified by comparing their chromosome locations. T-tests were used to compare the density of all variants and only indels among different regions of miRNA sequences or with that of the flanking regions.

### Indels in miRNA Target Sites

The start and end locations of 41 nt long PAR-CLIP footprints were obtained from Supplementary Table 7 of Hafner et al. [31]. We used the liftover tool in the Galaxy web-server [73] to convert the list of genomic locations presented in PAR-CLIP mRNA:miRNA interaction map to the GRCh37/hg19 assembly of the human genome. All variants and indels in the PAR-CLIP footprint regions were identified by comparing the locations of the footprints with the locations of variants in dbSNP build 135 downloaded from UCSC. We also identified all variants and indels in the 3′ UTRs of mRNAs using a similar procedure. Indels in the 3′ UTR of all mRNAs were also used to calculate the percentage of three indel classes, namely, single-base-pair, repeat expansions, and “other”. We also created a second list of indels that included only those indels in 3′ UTRs of mRNAs that are the experimentally supported target of at least one miRNA by collecting the mRNA-miRNA target pairs contained in miRecords [48], miTarBase [50], and TarBase 5.0 [49]. We used Galaxy [73] to extract 100 base pair long sequences surrounding the genomic location of each indel. From these 100 base pairs long sequences we made two set of sequences, the reference sequences and the mutation sequences that contained insertions and deletions. We then scanned these sequences to determine locations complementary to any of the six miRNA seed types as described by Ellwanger et al. [68]. Target sites found in the reference sequences but not in the mutant sequences, were marked as disrupted by the indel, while target sites found in the mutant sequences but not in the reference sequences were sites created by the indel. For indels within PAR-CLIP footprints, we determined how the indels created and disrupted putative target sites for any miRNA. For indels within 3′ UTRs of mRNAs in experimentally supported mRNA-miRNA pairs, we limited the search to only those miRNAs that have been found to target the mRNA. We also used indels from GATK resource bundle to find disrupted and created miRNA sites in PAR-CLIP footprint region and 3′UTRs of known miRNA target sites. T-tests were used to compare the density of all variants and only indels among different regions shown in Figure 2.

### Linking Indels with Pathway and Associations Studies

Genes with indels in the experimentally supported target sites of miRNAs were compared against the list of genes in KEGG pathways to select the five most enriched pathways using the DAVID annotation tools [74]. SNPs in experimentally supported miRNA target sites of the genes in these pathways were also identified using the same procedure for indels. KEGG Mapper, a tool for changing color schema in KEGG pathways was then used to represent genes with indels and/or SNPs in miRNA target sites [58].

Indels in miRNAs or experimentally supported target sites were linked with the results of association studies. High ranking markers for association studies were collected from dbGaP [75] and the NHGRI GWAS Catalog [60]. All indels in miRNAs or experimentally supported target sites within 100 kb of any of the these markers were then tested for linkage disequilibrium (LD) by using Haploview [76] software. In Haploview, we have selected the Gabriel et al [77] algorithm with its default parameter settings to define strong LD blocks. Confidence interval minima have been set with upper at 0.98 and lower at 0.7, while upper confidence interval maximum for strong recombination is set to 0.9 and all the markers with MAF value below 0.05 are excluded from block.

## Supporting Information

Figure S1 **Comparison of the percentage of indels that are single nucleotide indels, repeat expansions, or other types of indels among indels in miRNAs, 3′ UTRs, and the entire genome.**
(TIF)

Figure S2 **Genes in the cancer pathway containing SNPs and indels that altered experimentally supported target sites.** Genes containing only indels (pink), only SNPs (yellow), and both SNPs and indels (green) in target sites are within colored rectangles.
(TIF)

Figure S3 **Genes in the prostate cancer pathway containing SNPs and indels that altered experimentally supported target sites.** Genes containing only indels (pink), only SNPs (yellow), and both SNPs and indels (green) in target sites are within colored rectangles.
(TIF)

Figure S4 **Genes in the colorectal cancer pathway containing SNPs and indels that altered experimentally supported target sites.** Genes containing only indels (pink), only SNPs (yellow), and both SNPs and indels (green) in target sites are within colored rectangles.
(TIF)

Figure S5 **Genes in the ErbB signaling pathway containing SNPs and indels that altered experimentally supported target sites.** Genes containing only indels (pink), only SNPs (yellow), and both SNPs and indels (green) in target sites are within colored rectangles.
(TIF)

Table S1 List of indels in miRNAs.
(XLS)

Table S2 Indels in PAR-CLIP data found to disrupt or create sites for miRNA.
(XLS)

Table S3 Indels in the experimentally supported target sites for miRNA.
(XLS)

Table S4 Indels and SNPs from experimentally supported miRNA targets in KEGG pathway.
(XLS)
