# Supplementary figures and images for: Systematic Analysis of microRNA Targeting Impacted by Small Insertions and Deletions in Human Genome
Source: PLoS One. 2012 Sep 25;7(9):e46176. doi: 10.1371/journal.pone.0046176 (PMC3457991; doi:10.1371/journal.pone.0046176)

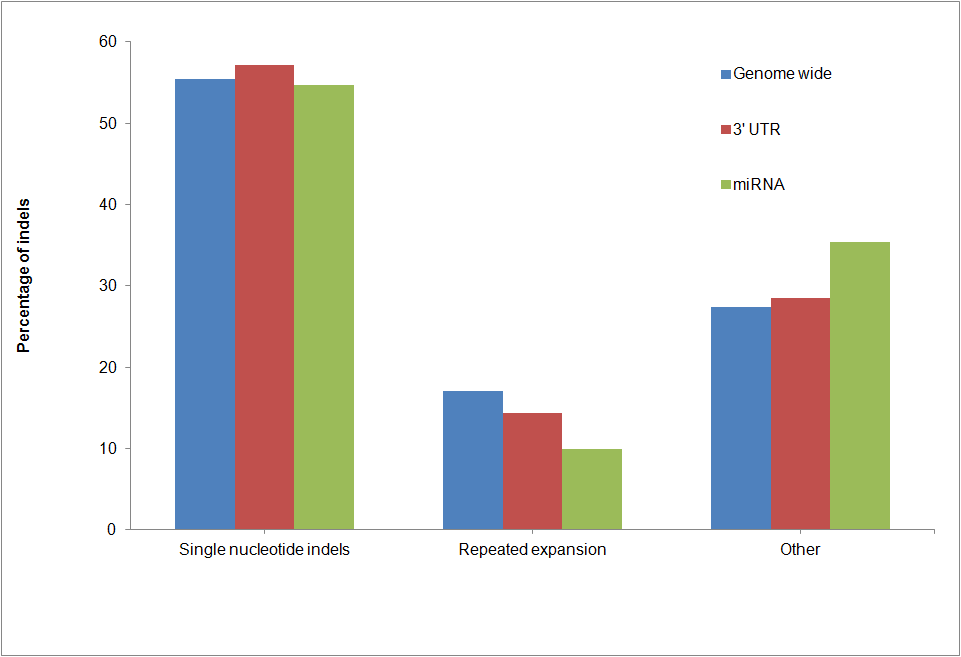

Supplement: Figure S1 — Comparison of the percentage of indels that are single nucleotide indels, repeat expansions, or other types of indels among indels in miRNAs, 3′ UTRs, and the entire genome. (TIF) [file pone.0046176.s001.tif]

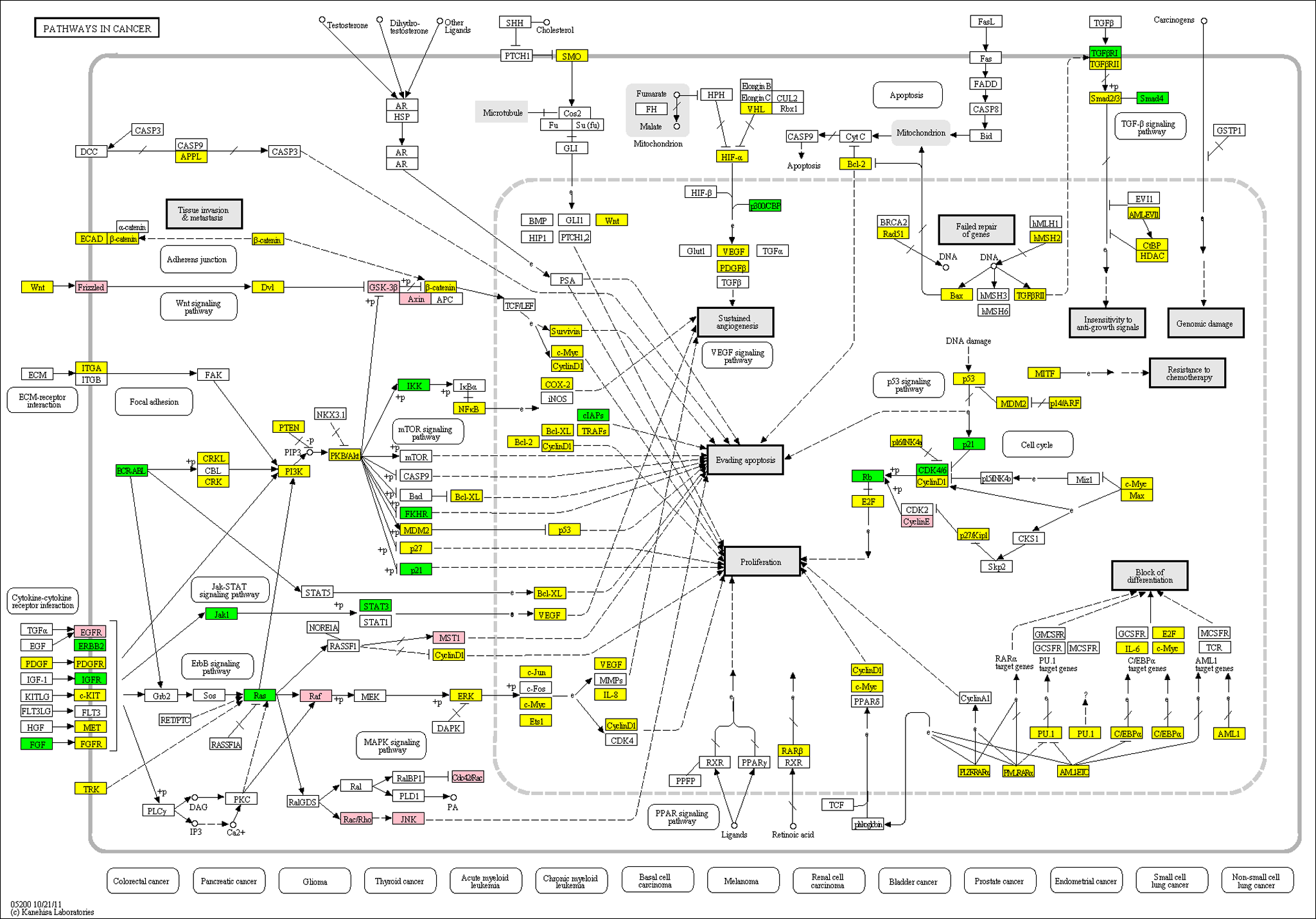

Supplement: Figure S2 — Genes in the cancer pathway containing SNPs and indels that altered experimentally supported target sites. Genes containing only indels (pink), only SNPs (yellow), and both SNPs and indels (green) in target sites are within colored rectangles. (TIF) [file pone.0046176.s002.tif]

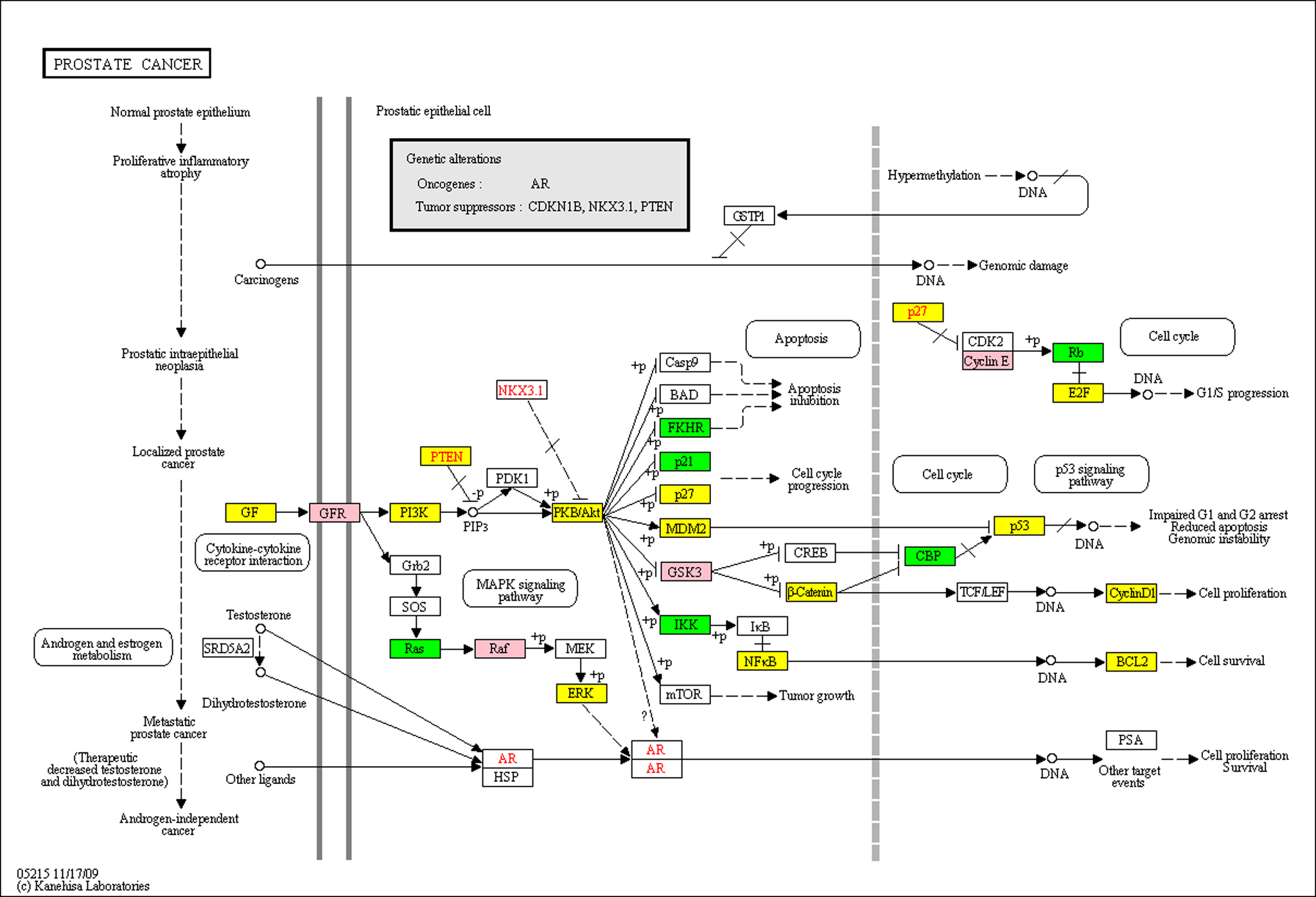

Supplement: Figure S3 — Genes in the prostate cancer pathway containing SNPs and indels that altered experimentally supported target sites. Genes containing only indels (pink), only SNPs (yellow), and both SNPs and indels (green) in target sites are within colored rectangles. (TIF) [file pone.0046176.s003.tif]

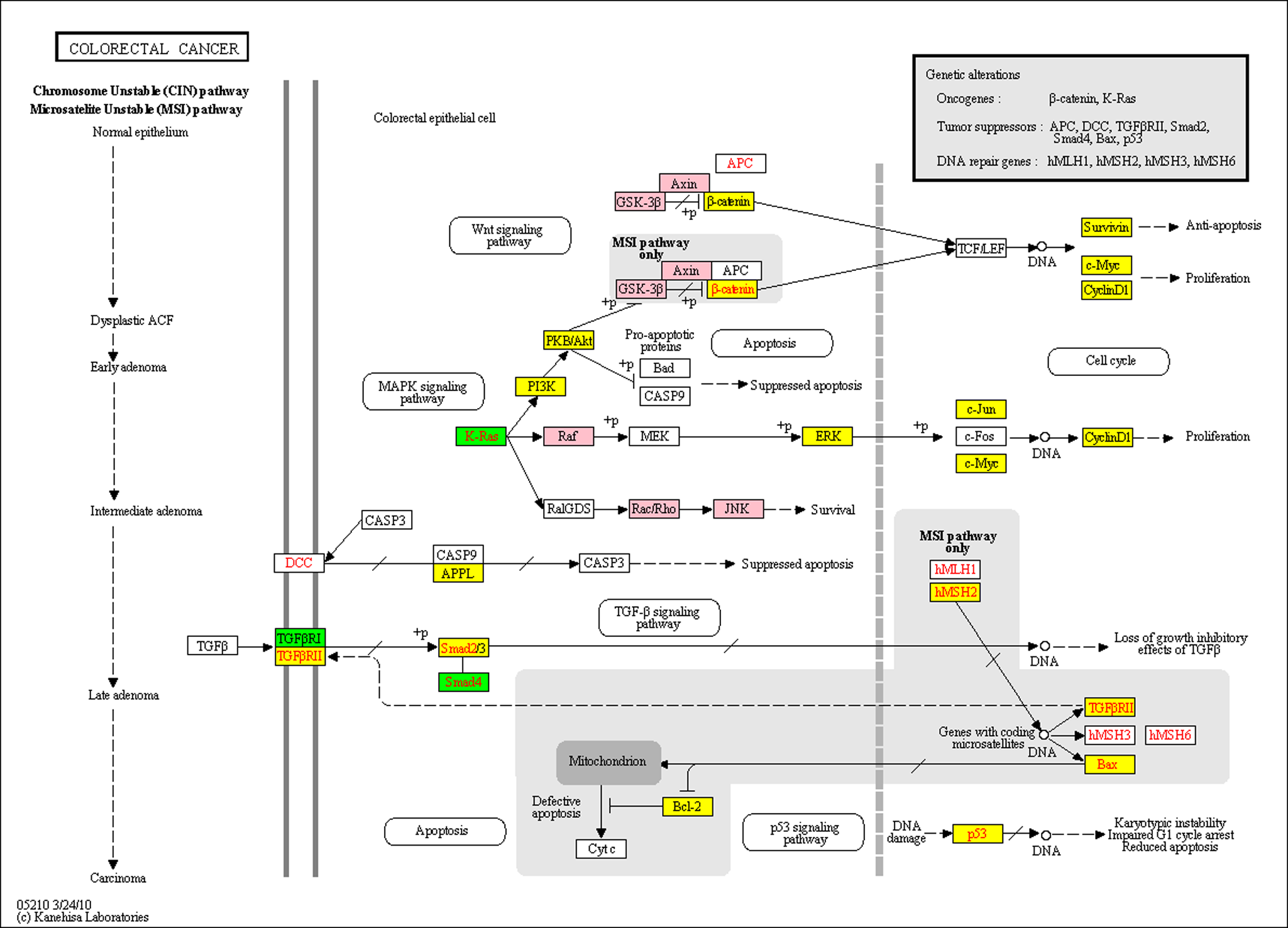

Supplement: Figure S4 — Genes in the colorectal cancer pathway containing SNPs and indels that altered experimentally supported target sites. Genes containing only indels (pink), only SNPs (yellow), and both SNPs and indels (green) in target sites are within colored rectangles. (TIF) [file pone.0046176.s004.tif]

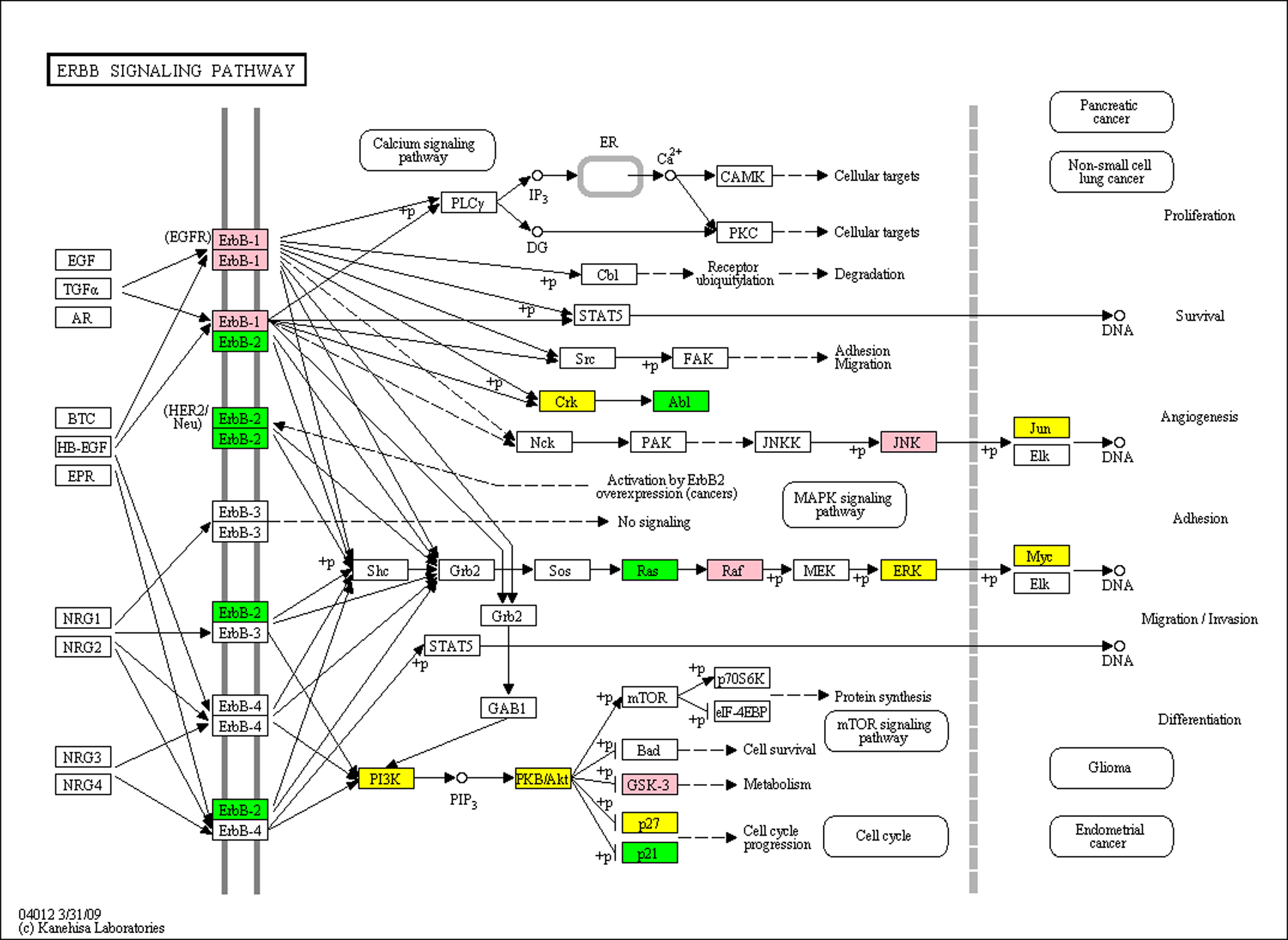

Supplement: Figure S5 — Genes in the ErbB signaling pathway containing SNPs and indels that altered experimentally supported target sites. Genes containing only indels (pink), only SNPs (yellow), and both SNPs and indels (green) in target sites are within colored rectangles. (TIF) [file pone.0046176.s005.tif]
